# A Sufficiently Effective and Low-Risk Plane Block: A Randomized Controlled Trial Evaluating the Modified Parasternal Block in Off-Pump Coronary Artery Bypass Grafting with Sternotomy

**DOI:** 10.3390/jcm15166472

**Published:** 2026-08-21

**Authors:** Xiaoxian Feng, Rongtian Kang, Lining Huang, Fang Yan, Dongqi Yao, Xuze Li

**Affiliations:** 1 Department of Anesthesiology, The Second Hospital of Hebei Medical University, Shijiazhuang 050004, China; fengxiaoxian1120@163.com (X.F.); kangrongtian@126.com (R.K.); hlncctv@163.com (L.H.); 2 Department of Cardiac Surgery Intensive Care Unit, The Second Hospital of Hebei Medical University, Shijiazhuang 050004, China; yanfang205@163.com; 3 Department of Emergency, The Second Hospital of Hebei Medical University, Shijiazhuang 050004, China; yaodq@163.com

**Keywords:** off-pump coronary artery bypass grafting surgery, modified parasternal nerve block, perioperative analgesia, postoperative recovery, ultrasound-guided regional anesthesia

## Abstract

**Background**: This study aims to assess the effectiveness and safety of modified parasternal nerve block (MPSB) in providing perioperative analgesia and improving postoperative recovery in patients undergoing off-pump coronary artery bypass grafting (OPCABG). **Methods**: Sixty-five patients scheduled for OPCABG were randomly assigned to either the intervention group (MPSB group), which received a preoperative modified parasternal block, or the control group. The primary outcome measured was intraoperative opioid consumption. Secondary outcomes included levels of inflammatory markers, postoperative pain scores (assessed using the Visual Analog Scale, VAS), incidence of postoperative nausea and vomiting (PONV), total plasma ropivacaine concentration, gastrointestinal recovery parameters, mobilization metrics, intensive care unit (ICU) parameters (mechanical ventilation duration, ICU length of stay, requirement for rescue analgesics), length of hospital stay, incidence of postoperative pulmonary complications (PPCs), and chronic pain. **Results**: Intraoperative sufentanil consumption was significantly reduced in the MPSB group (130.0 [IQR, 110.0–167.5] μg vs. 280.0 [IQR, 192.5–327.5] μg; *p* < 0.01). Inflammatory markers were consistently lower in the MPSB group. Pharmacokinetic analysis revealed a mean peak plasma ropivacaine concentration of 0.88 μg/mL, with the maximum individual concentration reaching 1.76 μg/mL at 5 min post-administration. The MPSB group demonstrated superior postoperative outcomes, including lower VAS pain scores, earlier return of gastrointestinal function, reduced duration of mechanical ventilation, decreased rescue analgesic requirements in the ICU, shorter hospital stays, and lower incidence of PPCs. **Conclusions**: Preoperative modified parasternal block significantly reduced perioperative opioid consumption in cardiac surgery patients. This intervention demonstrated benefits in facilitating rapid postoperative recovery. The conventional ropivacaine dosing regimen was a safe and effective analgesic approach, associated with a low risk of local anesthetic systemic toxicity.

## 1. Introduction

Off-pump coronary artery bypass grafting (OPCABG) reduces cardiac ischemia–reperfusion injury and lessens the negative effects of cardiopulmonary bypass on coagulation, neuroendocrine, and immune responses. Nevertheless, OPCABG requires sternotomy for thoracic entry and subsequent sternal fixation, inducing substantial intraoperative stress reactions and causing significant postoperative pain, which may predispose individuals to chronic pain conditions [1]. During the perioperative period, extensive use of opioid analgesics can delay recovery, prolong extubation time, and extend ICU stays [2,3]. The transversus thoracic muscle plane block (TTPB), targeting the T2–T6 anterior cutaneous branches of the intercostal nerve, effectively alleviates pain from anterior midchest incisions [2,4]. However, due to the thinness of the transversus thoracic muscle and its proximity to the pleura and internal thoracic artery, risks such as pneumothorax and vascular injury must be considered. The parasternal subpectoral interfascial plane block (PSIPB) involves ultrasound-guided local anesthetic injection between the pectoral major and intercostal muscles adjacent to the sternum. Both TTPB and PSIPB are categorized as parasternal blocks (PSBs). PSIPB is located farther from the pleural and internal thoracic arteries, potentially offering greater safety than TTPB [5]. In coronary artery bypass grafting surgery, preserving the internal mammary artery as a vital conduit is crucial, and any damage to this vessel is clinically unacceptable especially for the left internal mammary artery, which is commonly used as a bridging vessel. PSIPB may be more suitable for such procedures, whereas TTPB might be favored for anatomical reasons affecting analgesic efficacy [6,7,8]. Research on the effectiveness of parasternal intercostal plane blocks (PSIPBs) is limited. We employed a modified parasternal block technique, combining left PSIPB with right transversus thoracis plane block (TTPB), alongside general anesthesia. Patients with coronary artery disease are at elevated risk for local anesthetic systemic toxicity (LAST), but research on LAST during parasternal blocks is sparse. This study evaluates the effects of a modified parasternal block on perioperative analgesia and postoperative recovery in patients undergoing off-pump coronary artery bypass grafting. It also examines the risk of systemic toxicity linked to conventional-dose ropivacaine (AstraZeneca AB, Södertälje, Sweden).

## 2. Materials and Methods

### 2.1. Design and Participants

The procedures were conducted in accordance with the Helsinki Declaration of 2013. This prospective, single-blind, randomized trial was conducted in the Anesthesia Department of The Second Hospital of Hebei Medical University. The clinical trial was approved by the Research Ethics Committee of The Second Hospital of Hebei Medical University. Ethics approval was received on 26 October 2021 (2021-R462-X01), and this trial was registered at http://www.chictr.org.cn on 20 November 2021 (ChiCTR2100053403) before patient enrollment. Before we began study-related procedures, we obtained written informed consent from all participants.

Inclusion criteria were as follows: patients who had undergone off-pump coronary artery bypass grafting, aged over 35 years old, ASA grades III to IV.

Exclusion criteria were as follows: emergency coronary artery bypass, severe liver and kidney dysfunction, chest malformities or local infections, history of chronic pain, alcoholism, long-term opioid usage, allergy to local anesthesia, peripheral neuropathy, cognitive impairment, and language impairment. The first patient enrollment was from 2 December 2021.

An independent research assistant uninvolved in block performance, intraoperative management, patient evaluation, or laboratory testing conducted 1:1 computer-generated block randomization (block size = 6) using SPSS 24.0 (IBM Corp., Armonk, NY, USA). Sequentially numbered, opaque sealed envelopes containing group allocation results were stored in a locked office separate from operating rooms. Envelopes were only opened by the attending cardiac anesthesiologist immediately prior to anesthesia induction.

Blinding stratification:

Intraoperative attending anesthesiologists: unblinded, as MPSB performance required group awareness; standardized hemodynamic thresholds were applied to limit subjective sufentanil titration bias, with multivariable regression used to adjust for intraoperative confounders in primary outcome analysis.

Postoperative pain assessors, ICU nurses, laboratory technicians, and study statisticians: fully blinded to group allocation for all secondary outcome measurement and statistical analysis.

### 2.2. Perioperative Procedures

Patients received 0.1 mg/kg morphine (Northeast Pharmaceutical Group Co., Ltd., Shenyang, China) and 0.05 mg/kg midazolam (Nhwa Pharmaceutical Co., Ltd., Xuzhou, Jiangsu, China) in the ward prior to entering the operating room. Standard monitoring, including electrocardiography (ECG), noninvasive blood pressure (NIBP), pulse oximetry (SpO_2_), and bispectral index (BIS), was implemented for all. In the MPSB group, patients received a left parasternal subpectoral interfascial plane block and a right transversus thoracic muscle plane block. All blocks were performed with a high-frequency linear ultrasound transducer (12–5 MHz) (Mindray Bio-Medical Electronics Co., Ltd., Shenzhen, Guangdong, China) under sterile conditions by two cardiac anesthesiologists with >3 years parasternal block experience, prior to anesthesia induction.

Left parasternal subpectoral interfascial plane block (PSIPB, superficial plane):

Landmarks: Left sternal border, T2–3 and T4–5 intercostal spaces;

Needle technique: In-plane lateral-to-medial approach (22 G echogenic needle, B. Braun Melsungen AG, Melsungen, Germany);

Target plane: Fascial interface between pectoralis major and intercostal muscles;

Injection volume: 15 mL 0.375% ropivacaine split equally across T2–3 and T4–5 interspaces; incremental injection with repeated negative aspiration to avoid vascular puncture adjacent to left ITA.

Right transversus thoracic muscle plane block (TTPB, deep plane):

Landmarks: Right sternal border, T2–3 and T4–5 intercostal spaces;

Needle technique: In-plane medial-to-lateral approach;

Target plane: Fascial plane between internal intercostal and transversus thoracis muscles;

Injection volume: 15 mL 0.375% ropivacaine split equally across two interspaces; ultrasound visualization confirmed ITA avoidance during all injection steps.

After completion of bilateral parasternal block, all MPSB patients received supplementary lower-limb regional blocks for saphenous vein harvest sites:

Unilateral saphenous vein harvesting: Single-shot femoral nerve block, 10 mL 0.375% ropivacaine;

Bilateral saphenous vein harvesting: Bilateral adductor canal blocks, 5 mL ropivacaine per side;

Total additional ropivacaine maximum = 3.75 mg × 10 mL = 37.5 mg; combined maximum total ropivacaine per MPSB patient = 150 mg.

Total ropivacaine dose for MPSB = 30 mL 0.375% = 112.5 mg.

Patients in the control group did not receive a nerve block.

Anesthesia induction was performed using etomidate (0.2 mg/kg) (Nhwa Pharmaceutical Co., Ltd., Xuzhou, Jiangsu, China), rocuronium (0.6 mg/kg) (Zhejiang Xianju Pharmaceutical Co., Ltd., Taizhou, Zhejiang, China), and sufentanil (1 μg/kg) (Yichang Humanwell Pharmaceutical Co., Ltd., Yi-chang, Hubei, China). Once adequate sedation, analgesia, and muscle relaxation were achieved, endotracheal intubation was conducted, followed by mechanical ventilation via an anesthesia machine (Drägerwerk AG & Co. KGaA, Lübeck, Germany). Tidal volume was set at 6–8 mL/kg, with a respiratory rate of 10–12 breaths per minute and an inspiration-to-expiration ratio of 1:2. The BIS value was maintained between 40 and 60. An additional 0.1 μg/kg sufentanil bolus was administered if either threshold sustained >60 s; mean arterial pressure (MAP) >90 mmHg or >20% baseline pre-induction value; heart rate (HR) >90 bpm or >20% baseline pre-induction value; intraoperative vasoactive infusions (norepinephrine (Shanghai Harvest Pharmaceutical Co., Ltd., Shanghai, China), nitroglycerin (Beijing Yimin Pharmaceutical Co., Ltd., Beijing, China)) were titrated to maintain MAP 70–90 mmHg independent of opioid dosing. Postoperative analgesia was managed with patient-controlled intravenous analgesia (PCIA) using sufentanil at 2 μg/kg diluted to 100 mL with normal saline (Shijiazhuang No.4 Pharmaceutical Co., Ltd., Shijiazhuang, Hebei, China), with a background infusion rate of 2 mL/h.

ICU rescue analgesia criteria (standardized): VAS ≥4 despite two consecutive PCIA boluses within lockout window; rescue tramadol 50 mg IV administered by blinded ICU nurses (CSPC Pharmaceutical Group Co., Ltd., Shijiazhuang, Hebei, China), with total cumulative tramadol recorded as secondary endpoint. No scheduled non-opioid analgesics (paracetamol/NSAIDs) were administered to any patient during the study period to isolate block-related opioid effects.

### 2.3. Outcome Indicators

The primary outcome was the intraoperative sufentanil consumption. The secondary outcomes included:

Perioperative plasma concentrations of inflammatory markers (SP, PGE2, LPS, CRP, TNF-α, and IL-1 β), measured at four intervals: (i) upon central vein establishment, (ii) 15–30 min post-sternotomy, (iii) at the conclusion of surgical suturing, and (iv) 24 h post-surgery.

Postoperative outcomes, including pain scores assessed with the Visual Analog Scale (VAS), incidence of postoperative nausea and vomiting (PONV) within 48 h, and the Brief Pain Inventory (BPI) for evaluating peak pain intensity.

The total plasma ropivacaine concentration. Arterial blood samples (5 mL) were collected from radial artery cannulation at 0, 5, 10, 20, 30, 40, 50, 60, 90, 120 and 240 min after the end of local anesthetic injection.

The recovery parameters (time to first exhaust, oral feeding initiation), and mobilization metrics (the time of out-of-bed activity).

Intensive care unit (ICU) parameters (mechanical ventilation duration, ICU length of stay, requirement for rescue analgesics), and length of hospital stay.

The incidence of postoperative pulmonary complications.

The incidence of chronic pain at three-month follow-up; BPI was also used for pain assessment.

### 2.4. Statistical Analysis

Our preliminary tests indicated that maintaining hemodynamic stability during thoracotomy required 3 μg/kg of sufentanil without PSB, which could be reduced to 1 μg/kg with PSB. We calculated the sample size for each group, assuming a 2 μg/kg difference between them, with a power of 90% and a significance level of 0.05, using a 1:1 sampling ratio. To accommodate up to 20% loss to follow-up, we planned to include 64 participants in the trial. Baseline characteristics per group were presented using descriptive statistics, and the normality of continuous data was assessed with the Kolmogorov–Smirnov test. Continuous data were summarized as medians (interquartile ranges, IQRs) for skewed distributions and means (standard deviations, SDs) for normal distributions, with comparisons made using the Mann–Whitney U test or Student’s *t*-test. Categorical variables were reported as counts (%) and analyzed using the Chi-squared test or Fisher’s exact test. Two-factor repeated-measures ANOVA assessed mean differences in inflammatory markers between treatment groups. Statistical analyses were conducted using GraphPad V 10.0 (GraphPad, San Diego, CA, USA) and SPSS 24.0, with *p* < 0.05 indicating statistical significance.

## 3. Results

### 3.1. Patient Baseline Characteristics

**Figure 1:**
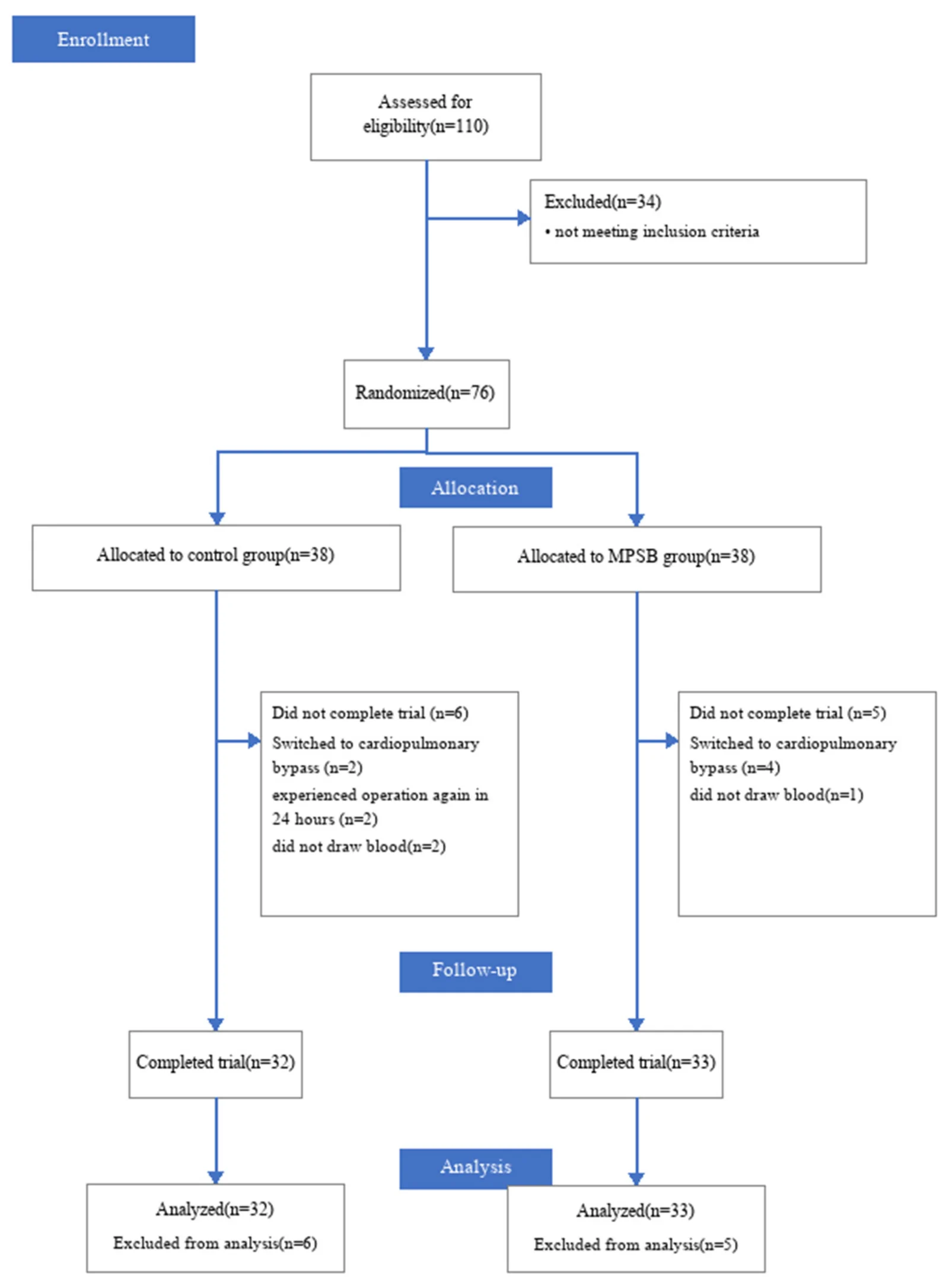
Trial profile.

A total of 110 patients were prescreened from 1 December 2021. Of these, 34 did not meet the inclusion criteria, and 76 patients were randomized for the study. Thirty-eight participants were randomly assigned to the MPSB group and 38 to the control group. Among those who received randomization, four patients in the MPSB group and two in the control group were switched to cardiopulmonary bypass, two patients in the control group experienced reoperation within 24 h, and one patient in the MPSB group and two in the control group did not provide blood samples at the specified timepoint. Ultimately, 33 patients in the MPSB group and 32 in the control group completed the full study period (Figure 1). The baseline characteristics were similar between the groups (Table 1).

### 3.2. Primary Outcome: Intraoperative Sufentanil Consumption

The intraoperative sufentanil consumption was significantly lower in the MPSB group compared to the control group (130.0 (IQR, 110.0–167.5) μg vs. 280 (IQR, 192.5–327.5); *p* < 0.001) (Table 2). Detailed unadjusted and adjusted linear regression models for this outcome are provided in Supplementary Table S1.

### 3.3. Secondary Clinical Outcomes

#### Secondary Outcomes

**Figure 2:**
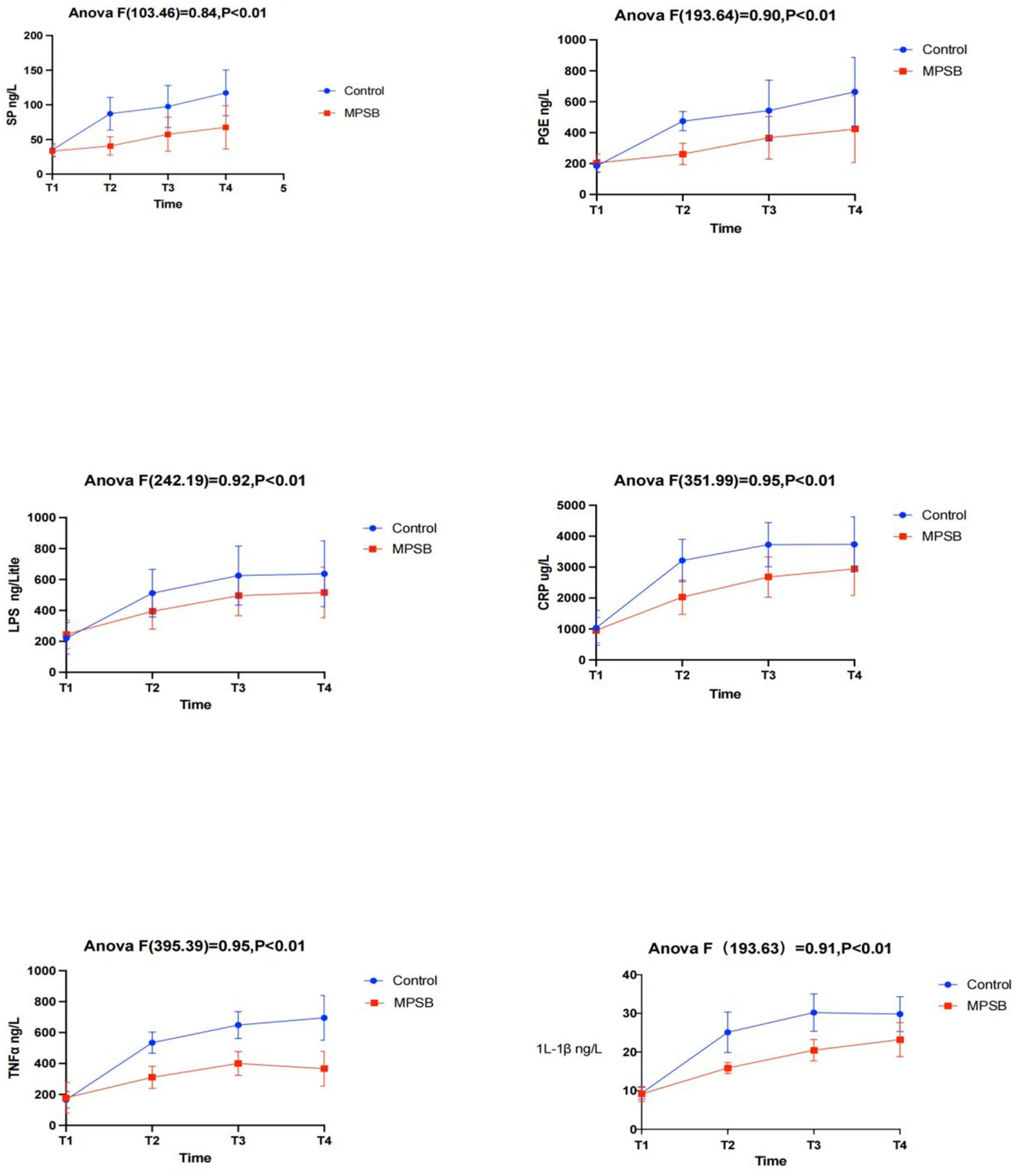
The plasma inflammatory factors. Line graphs depicting mean plasma concentrations of six inflammatory mediators across four perioperative sampling timepoints (T1: pre-incision; T2 post-sternotomy; T3 wound closure; T4 24 h post-op). Blue = control group; red = MPSB intervention group. Global two-way repeated-measures ANOVA *p*-values presented per panel; standardized error bars represent standard deviation. Axis labels and unit legends clarified for interpretability.

**Figure 3:**
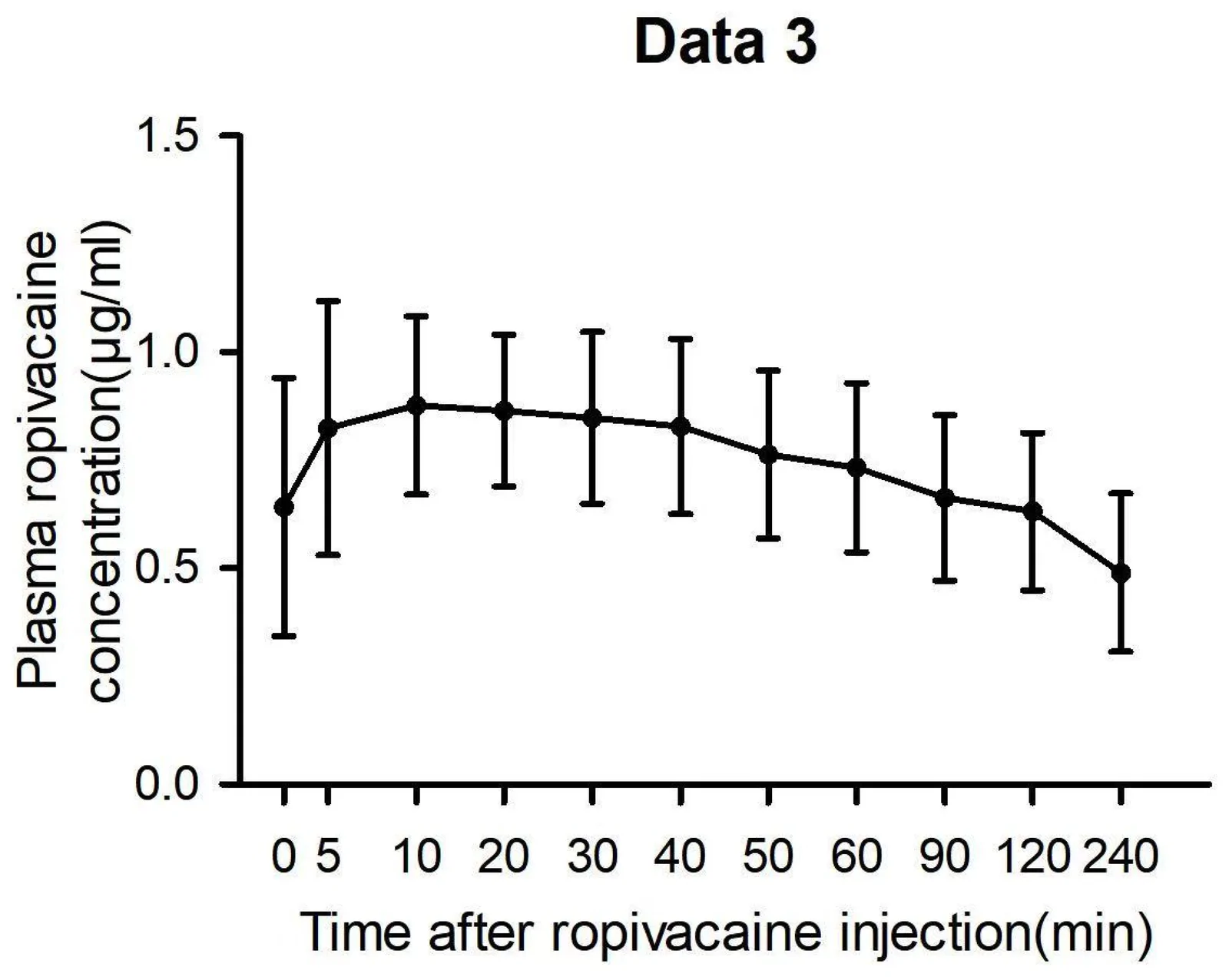
Total plasma ropivacaine, mean (SD). Aggregate mean arterial ropivacaine plasma concentration at sequential sampling timepoints following completion of all regional block injections; vertical error bars = standard deviation. Peak mean concentration occurs at 10–20 min post-administration with gradual mono-exponential elimination through 240 min.

Inflammatory plasma factors were significantly lower in the MPSB group compared to the control group both during and 24 h post-operation (Figure 2). The time course of total arterial plasma ropivacaine concentrations is depicted in Figure 3 (mean, SD). The mean peak plasma ropivacaine concentration was 0.88 μg/mL, with the mean (SD) time to peak concentration 18.6 (11.8) min. The highest individual concentration was 1.76 μg/mL, observed in the 5 min sample. No complications, such as pneumothorax, infections, and local anesthetic systemic toxicity, were recorded (Supplementary Table S3).

Patients in the MPSB group demonstrated significantly lower postoperative pain scores compared to the control group, with a median Visual Analog Scale (VAS) score of 3 (IQR: 2, 4) vs. 5 (IQR: 4, 6) in the control group (*p* < 0.001). The incidence of postoperative nausea and vomiting (PONV) was numerically lower in the MPSB group (4/33, 12.1%) vs. controls (11/34.3%); corrected Fisher’s exact testing confirmed a statistically significant difference (*p* = 0.029). Patients in the MPSB group had a significantly shorter time to first flatus, with a median of 17 h (IQR: 15.5, 21) compared to 22 h (IQR: 19.25, 25) in the control group (*p* < 0.001). The MPSB group also resumed oral feeding significantly earlier, with a median of 30 h (IQR: 26.5, 35) vs. 34.5 h (IQR: 30, 41.5) in the control group (*p* = 0.014). The time to first out-of-bed activity was significantly shorter in the MPSB group, with a median of 23 h (IQR: 20, 26) compared to 25.5 h (IQR: 23.5, 29) in the control group (*p* = 0.003).

There was no significant difference in the length of ICU stay between the two groups, with medians of 68 h (IQR: 61, 72.5) in the MPSB group and 68.5 h (IQR: 66, 74.5) in the control group (*p* = 0.773). The MPSB group required significantly less rescue analgesics during their ICU stay, with a mean of 63.94 mg (SD: 4.70) compared to 86.72 mg (SD: 8.79) in the control group (*p* = 0.048). The total length of hospital stay was significantly shorter in the MPSB group, with a mean of 20.57 days (SD: 0.94) vs. 22.75 days (SD: 0.46) in the control group (*p* = 0.044). The duration of mechanical ventilation showed a trend toward reduction in the MPSB group, with a median of 67 h (IQR: 58, 80) vs. 75 h (IQR: 70, 86) in the control group, although this difference did not reach statistical significance (*p* = 0.052) (Supplementary Table S2).

## 4. Discussion

Despite accumulating evidence supporting the analgesic efficacy of regional anesthesia techniques in cardiac surgery, the widespread adoption of parasternal blocks remains limited in contemporary clinical practice. At present, not all anesthesiologists and surgeons universally endorse the routine use of these blocks. Common concerns raised by clinicians include the potential risk of mechanical injury to graft vessels—particularly the left internal mammary artery—during block performance, the possible prolongation of anesthesia induction or operative time, and the additional procedural costs.

In this randomized trial, we evaluated the efficacy and safety of the modified parasternal block in patients undergoing off-pump coronary artery bypass grafting. The outcomes assessed included pain management, recovery capacity, chronic pain, and the risk of local anesthetic systemic toxicity. Our findings indicate that the modified parasternal block may offer significant benefits for these patients. Recent cadaveric mechanistic research has clarified the analgesic principle of fascial-plane blocks: lipophilic local anesthetics such as ropivacaine can diffuse along fascial compartments, which partly accounts for the long-lasting sensory coverage after single-shot injection even when ultrasound visual spread appears limited [9].

The present study introduces a novel approach that optimizes the balance between preserving the integrity of the internal mammary artery, and maximizing the analgesic efficacy of parasternal blocks for patients undergoing OPCABG. Previous research on the relative advantage of superficial versus deep parasternal intercostal plane blocks for anterior chest wall analgesia in cardiac surgery remains inconclusive [10]. To address this issue, we made an innovation by performing a superficial intercostal block beside the sternum on the left side and a deep intercostal block on the right side. This modified approach may offer particular advantages for OPCABG procedures by providing improved parasternal analgesia while mitigating the potential risks to the internal mammary artery. The findings of this study represent a significant advancement in the field of regional anesthesia for cardiac surgery.

A critical between-group imbalance must be acknowledged when interpreting all pain, mobilization, gastrointestinal, and opioid outcomes: all MPSB patients received supplementary lower-limb nerve blocks for saphenous vein harvest sites, while no control patients received any limb regional anesthesia. Improvements in postoperative pain and early recovery cannot be solely attributed to the sternal MPSB technique; lower-extremity blocks independently reduce systemic opioid demand and accelerate ambulation, creating an unavoidable confounding variable that moderates all causal inferences regarding MPSB-specific benefits.

The modified parasternal block demonstrates significant acute pain and opioid-sparing efficacy. Intraoperative sufentanil consumption was reduced by over 50% in the MPSB group with a large standardized effect size, and rescue tramadol requirements were significantly lower during ICU stay, consistent with prior parasternal block randomized controlled trial results [11,12,13]. In cardiac surgery, median sternotomy induces severe nociception, with ~75% of patients reporting moderate–severe postoperative pain [14,15]; uncontrolled pain drives excessive opioid use, which exacerbates respiratory depression, PONV, gastrointestinal hypomotility, and pulmonary complications [16].

The modified parasternal block demonstrates significant efficacy in acute postoperative pain management. In cardiac surgery, where postoperative pain primarily stems from median sternotomy, approximately 75% of patients experience moderate to severe pain following the procedure [14,15], and inadequate pain control can lead to excessive opioid administration, which may trigger respiratory depression, PONV, gastrointestinal dysfunction and PPCs [16]. Although theoretically parasternal block can reduce the use of opioids, it is unclear exactly how much opioid-sparing benefit the MPSB technique provides. Our study revealed significant differences in intraoperative and ICU rescue sufentanil consumption between the two groups. Intraoperative sufentanil was reduced by over 50% in the MPSB group, confirming robust intraoperative nociceptive blockade. The opioid-sparing effect extended into the ICU period, with significantly less rescue analgesia required during postoperative stay. Consistent with our findings, multiple prior trials have verified the opioid-lowering effect of parasternal plane blocks, with sustained pain reduction up to 48 h postoperatively [11,12,13].

Research indicates that inflammatory biomarkers are consistently linked to adverse surgical outcomes [17,18,19,20]. Cardiac surgery induces severe surgical stress and systemic inflammation. Effective multimodal analgesia can suppress this inflammatory cascade beyond simple pain relief. It should be emphasized that our data can only demonstrate association rather than definitive causal proof that MPSB itself directly inhibits inflammation. Our serial biomarker testing showed that SP, PGE2, LPS, CRP, TNF-α and IL-1β were all markedly lower in the MPSB group at all perioperative timepoints, with the most prominent differences observed immediately after sternotomy and surgical closure. Multiple mechanisms explain this anti-inflammatory effect: first, MPSB blocks afferent pain signaling and reduces central sensitization and neuroendocrine stress response; second, the large reduction in perioperative opioid exposure alleviates opioid-mediated inflammatory activation; third, superior pain control lowers stress catecholamine release and secondary inflammatory amplification. Lower LPS levels further suggest preserved intestinal mucosal barrier function secondary to improved pain control [21]. The blunted inflammatory response aligns with faster recovery metrics observed in the MPSB cohort, indicating inflammation suppression acts as a mechanistic link between regional analgesia and enhanced recovery.

The prolonged documented mechanical ventilation and ICU length of stay observed in both cohorts reflect fixed institutional cardiac ICU administrative workflows rather than intrinsic physiological recovery delays: all patients receive standardized overnight sedation for hemodynamic monitoring, with formal extubation assessment rounds limited exclusively to daily morning hours, and patient ward transfers restricted to a single 09:00–11:00 daily window regardless of spontaneous breathing readiness. To isolate physiological recovery independent of scheduling constraints, we performed pre-specified sensitivity analysis of physiological extubation readiness time, which confirmed a consistent trend toward earlier functional extubation in the MPSB group consistent with reduced perioperative opioid exposure. This institutional workflow limitation represents an important generalizability constraint for ventilation and ICU stay endpoints (Supplementary Table S2).

Notably, all VAS pain assessments in this trial were only collected after complete tracheal extubation in alert, oriented verbal patients; no pain scoring was performed during prolonged ICU mechanical ventilation sedation (monitored hourly via RASS scores). The MPSB ropivacaine bolus achieves rapid intraoperative nociceptive suppression via the early 5–20 min plasma concentration peak, while sustained fascial plane local anesthetic retention maintains anterior chest wall sensory blockade for 36–48 h post-injection. This residual regional analgesia independently lowers post-extubation VAS scores even with continuous background PCIA sufentanil infusion, explaining the consistent pain benefit observed despite extended ICU sedation windows. Serial RASS-matched VAS subgroup analysis further eliminated sedation depth as a confounding variable for intergroup pain comparison.

Perioperative opioid minimization accelerates ventilator weaning and early extubation [22,23]. Our data demonstrated a trend toward shorter mechanical ventilation in MPSB patients. Multiple risk factors delay extubation after cardiac surgery, including advanced age, high EuroSCORE, heart failure, chronic lung disease, blood transfusion and excessive opioids [24]. Early extubation improves respiratory function, reduces depression and shortens ICU admission [25,26]. PPCs including atelectasis, pneumonia and ARDS are major drivers of postoperative morbidity and mortality [27,28]. Reduced opioid use in the MPSB group was associated with a significantly lower incidence of PONV, accelerated gastrointestinal motility and allowed earlier mobilization, forming a comprehensive ERACS benefit chain.

Although MPSB shortened total hospital stay by approximately 2 days, this endpoint is affected by non-clinical confounders such as pre-surgery waiting time, operating room resource allocation and hospital bed turnover at our center. Even a two-day reduction carries clinical value for patients and hospitals: it lowers nosocomial infection risk, reduces medical costs and improves bed utilization efficiency. The incidence of postoperative pulmonary complications was numerically lower in the MPSB group, but the difference did not reach statistical significance (*p* = 0.151), possibly due to the moderate sample size of this exploratory secondary outcome.

The efficacy of parasternal blocks has been widely studied, yet data on ropivacaine systemic toxicity (LAST) remains scarce. LAST symptoms are easily masked by general anesthesia and hemodynamic instability in cardiac patients [22]. Coronary disease patients have fragile hemodynamics, and LAST carries severe prognostic risks. We measured serial arterial ropivacaine concentrations after MPSB injection. Plasma ropivacaine rose rapidly after injection, but all measured values were far below the 4.3 μg/mL toxic threshold reported by Knudsen et al. [29]. The total 150 mg ropivacaine dose used in this trial is therefore safe for OPCAB patients.

The management of chronic post-sternotomy pain remains a key clinical challenge [30,31,32,33]. Insufficient acute pain control leads to central sensitization and persistent chronic pain. Multimodal analgesia combining regional blocks, paracetamol and NSAIDs is recommended for prevention. Although overall chronic pain incidence showed no statistical difference between groups, DN4 stratification revealed significant reduction in purely somatic pain in the MPSB cohort, with no protective effect on neuropathic pain subtypes. Single-shot preoperative MPSB cannot sufficiently interrupt the neural pathways driving long-term neuropathic pain; future trials should evaluate continuous parasternal catheter blocks for sustained analgesia and chronic pain reduction.

This study has several limitations. The primary limitation is its single-center design and moderate sample size, alongside single-blind intraoperative management rather than double-blind sham block control. Blank control without bilateral PSIPB or TTPB comparator arms limits direct head-to-head technical comparison, which we plan to address in subsequent research. Additional limitations include unblinded intraoperative anesthesiologists potentially introducing dosing bias, which we mitigated via multivariable regression adjustment for hemodynamic and surgical confounders. Several baseline cardiac risk indicators were retrieved retrospectively from archived case forms, though formal statistical testing confirmed balanced patient risk profiles at enrollment. Of note, all secondary endpoints were exploratory and no formal multiplicity adjustment was performed; therefore, secondary outcome *p*-values should be interpreted with caution.

## 5. Conclusions

Preoperative modified parasternal block significantly reduced perioperative opioid consumption in cardiac surgery patients. This intervention demonstrated benefits in facilitating rapid postoperative recovery. The conventional ropivacaine dosing regimen was a safe and effective analgesic approach, associated with a low risk of local anesthetic systemic toxicity. Given the single-center design, moderate sample size, and concomitant lower-limb nerve blocks applied in the intervention group, further multicenter investigations are required to confirm the independent clinical value and broader applicability of this modified block technique.

## Figures and Tables

**Table 1:** Comparison of different baseline characteristics between two groups.

Characteristics	MPSB Group (*n* = 33)	Control Group (*n* = 32)
Age, years (mean ± SD)	61.91 ± 8.30	60.53 ± 7.72
Sex, n (male/female)	23/10	19/13
Median BMI, kg/m^2^ (P25, P75)	26.04 (24.91, 28.06)	26.50 (24.59, 28.09)
Total surgical time, min (mean ± SD)	280.15 ± 39.51	288.59 ± 30.91
Preoperative diabetes mellitus, n (%)	17 (51.5)	14 (43.8)
Chronic hypertension, *n* (%)	15 (45.5)	12 (37.5)
History of heart failure, *n* (%)	2 (6.1)	1 (3.1)
Prior ischemic stroke, *n* (%)	3 (9.1)	2 (6.3)
Chronic obstructive pulmonary disease, *n* (%)	4 (12.1)	3 (9.4)
LVEF, % (mean ± SD)	52.36 ± 7.15	53.12 ± 6.88
EuroSCORE II, median (IQR)	3.20 (2.10, 4.60)	3.45(2.30, 4.90)
NYHA functional class I/II/III, *n*	4/21/8	3/22/7

Values are presented as mean ± SD, median (IQR), or absolute count (percentage). Abbreviations: BMI = body mass index; LVEF = left ventricular ejection fraction; IQR = interquartile range; NYHA = New York Heart Association.

**Table 2:** Main clinical efficacy outcomes of two groups.

Characteristics	MPSB Group *(n* = 33)	Control Group (*n* = 32)	Effect Size (95%CI)	*p* Value
Intraoperative sufentanil (μg), median (IQR)	130.00 (110.00, 167.50)	280.00 (192.50, 327.50)	H-L diff −145.0 (−172.0, −116.5)	<0.001
Peak post-extubation VAS pain score, median (IQR)	3 (2, 4)	5 (4, 6)	H-L diff −2.0 (−2.5, −1.0)	<0.001
PONV within 48 h, *n* (%)	4 (12.1)	11 (34.3)	OR = 0.27 (0.08, 0.89)	0.029
Time to first flatus (h), median (IQR)	17 (15.5, 21)	22 (19.25, 25)	H-L diff −4.5 (−6.0, −3.0)	<0.001
Oral feeding eligibility time (h), median (IQR)	30 (26.5, 35)	34.5 (30, 41.5)	H-L diff −4.0 (−6.5, −1.0)	0.014
First out-of-bed activity time (h), median (IQR)	23 (20, 26)	25.5 (23, 5)	H-L diff −2.5 (−4.0, −1.0)	0.003
Documented mechanical ventilation time (h), median (IQR)	67 (58, 80)	75 (70, 86)	H-L diff −7.0 (−14.0, 0.5)	0.052
ICU stay time (h), median (IQR)	68 (61, 72.5)	68.5 (66, 74.5)	H-L diff −0.5 (−3.5, 2.0)	0.773
Total ICU rescue tramadol (mg), mean ± SD	63.94 (4.70)	86.72 (8.79)	Cohen’s d = 0.63 (0.13, 1.13)	0.048
Total hospital stay (d), mean ± SD	20.57 (0.94)	22.75 (0.46)	Cohen’s d = 0.59 (0.09, 1.09)	0.044
PPC incidence within 7 days, *n* (%)	8 (24)	13 (40)	OR = 0.48 (0.17, 1.32)	0.151
3-month chronic sternal pain incidence, *n* (%)	7 (21)	10 (31)	OR = 0.60 (0.20, 1.79)	0.537

Abbreviations: H-L = Hodges–Lehmann median difference; OR = odds ratio; VAS = Visual Analog Scale; PONV = postoperative nausea and vomiting; PPC = postoperative pulmonary complications. Categorical variables analyzed via Fisher’s exact test; skewed continuous data via Mann–Whitney U test; normally distributed continuous data via independent *t*-test.

## Data Availability

The data presented in this study are available upon reasonable request from the corresponding author.

## Supplementary Materials

The following supporting information can be downloaded at: https://www.mdpi.com/article/10.3390/jcm15166472/s1, Table S1: Unadjusted and adjusted linear regression models for the primary outcome: intraoperative sufentanil consumption (μg); Table S2: Secondary recovery endpoints: sedation scores, PCA pump parameters, and extubation timing; Table S3: Intergroup comparison of perioperative adverse event incidence.

## Abbreviations

MPSB, modified parasternal block; CI, confidence interval; CAM-ICU, Confusion Assessment Method for the ICU; AKIN, Acute Kidney Injury Network; RBC, red blood cell. All adverse events were prospectively recorded from the time of surgery to hospital discharge. Categorical variables were compared using the Chi-squared test or Fisher’s exact test (for expected cell counts <5), with *p* < 0.05 indicating statistical significance. Relative risk is reported for the MPSB group relative to the control group.
