# Supplementary material for: A Sufficiently Effective and Low-Risk Plane Block: A Randomized Controlled Trial Evaluating the Modified Parasternal Block in Off-Pump Coronary Artery Bypass Grafting with Sternotomy
Source: J Clin Med. 2026 Aug 21;15(16):6472. doi: 10.3390/jcm15166472 (PMC13513453; doi:10.3390/jcm15166472)
Supplement: Supplementary file 1 [file jcm-15-06472-s001.zip › jcm-4462863-supplementary.pdf]

Supplementary Table S1. Unadjusted and adjusted linear regression models for the primary outcome: intraoperative sufentanil consumption (μg)

**Variable**	**Unadjusted Model**			**Adjusted Model**		
	Regression Coefficient (β)	95% Confidence Interval	P-value	Regression Coefficient (β)	95% Confidence Interval	P-value
Group allocation (MPSB=1, Control=0)	-150.00	-182.37 to -117.63	<0.001	-142.71	-176.84 to -108.58	<0.001
Total intraoperative vasoactive agent dosage (μg/kg)	0.02	0.01 to 0.03	0.021	0.01	0.00 to 0.02	0.068
Baseline mean arterial pressure (mmHg)	1.23	0.12 to 2.34	0.031	0.98	-0.15 to 2.11	0.088
Baseline heart rate (bpm)	0.87	0.21 to 1.53	0.011	0.72	0.06 to 1.38	0.033
Total surgical duration (min)	0.32	0.11 to 0.53	0.003	0.28	0.07 to 0.49	0.009
Number of coronary bypass grafts	12.45	3.21 to 21.69	0.008	10.87	1.54 to 20.20	0.023
Model R^2	0.52	—	<0.001	0.61	—	<0.001

Abbreviations: MPSB, modified parasternal block; CI, confidence interval. Adjusted model controls for all covariates listed in the table. The unadjusted model only includes group allocation as the independent variable. Intraoperative sufentanil consumption was defined as total cumulative sufentanil from anesthesia induction to sternal skin closure. All statistical analyses were performed using SPSS 24.0, with p<0.05 indicating statistical significance.

Supplementary Table S2. Secondary recovery endpoints: sedation scores, PCA pump parameters, and extubation timing

Outcome Metric	MPSB Group (n=33)	Control Group (n=32)	P-value
Hourly RASS sedation score during mechanical ventilation, median (IQR)			
0–6 h post-ICU admission	-3 (-4, -2)	-3 (-4, -2)	0.742
6–12 h post-ICU admission	-2 (-3, -1)	-2 (-3, -1)	0.689
12–24 h post-ICU admission	-1 (-2, 0)	-1 (-2, 0)	0.815
24–48 h post-ICU admission	0 (-1, 1)	0 (-1, 1)	0.773
PCA pump parameters, median (IQR)			
Total PCA demand attempts	12 (8, 17)	21 (15, 28)	<0.001
Total successful PCA bolus deliveries	9 (6, 13)	16 (11, 22)	<0.001
Extubation timing endpoints, median (IQR), hours			
Documented mechanical ventilation duration	67 (58, 80)	75 (70, 86)	0.052
Physiological extubation readiness time	42 (36, 48)	48 (42, 54)	0.027

Abbreviations: RASS, Richmond Agitation-Sedation Scale; PCA, patient-controlled intravenous analgesia; MPSB, modified parasternal block; IQR, interquartile range. RASS scores: -5 = unarousable, -4 = deep sedation, -3 = moderate sedation, -2 = light sedation, -1 = drowsy, 0 = alert and calm. Physiological extubation readiness time was defined as the time from ICU admission to meeting standard extubation criteria (spontaneous breathing, $FiO_2 \leq 0.4$, PEEP ≤5 cmH_2O, stable hemodynamics), independent of institutional ICU extubation scheduling rules. Continuous variables were compared using the Mann-Whitney U test, with $p<0.05$ indicating statistical significance.

Supplementary Table S3. Intergroup comparison of perioperative adverse event incidence

**Adverse Event**	**MPSB Group (n=33)**	**Control Group (n=32)**	**Relative Risk (95% CI)**	**P-value**
Unplanned ICU readmission, n (%)	1 (3.0)	2 (6.3)	0.485 (0.046, 5.171)	0.612
Pericardial effusion requiring drainage, n (%)	0 (0.0)	1 (3.1)	0.323 (0.013, 7.715)	0.492
New-onset arrhythmia (atrial/ventricular), n (%)	3 (9.1)	5 (15.6)	0.582 (0.151, 2.247)	0.471
ICU delirium (CAM-ICU positive), n (%)	1 (3.0)	4 (12.5)	0.242 (0.028, 2.061)	0.198
Postoperative clinical stroke, n (%)	0 (0.0)	0 (0.0)	—	1.000
Acute kidney injury (AKIN stage ≥1), n (%)	2 (6.1)	3 (9.4)	0.646 (0.114, 3.659)	0.671
All-cause in-hospital mortality, n (%)	0 (0.0)	0 (0.0)	—	1.000
Surgical re-exploration for hemorrhage, n (%)	1 (3.0)	2 (6.3)	0.485 (0.046, 5.171)	0.612
Packed red blood cell transfusion, n (%)	7 (21.2)	10 (31.3)	0.679 (0.295, 1.564)	0.347
Total RBC transfusion volume, mL, median (IQR)	200 (0, 400)	300 (0, 600)	—	0.219
